# Immunotherapy in Prostate Cancer: State of Art and New Therapeutic Perspectives

**DOI:** 10.3390/curroncol30060432

**Published:** 2023-06-13

**Authors:** Felicia Maria Maselli, Francesco Giuliani, Carmelo Laface, Martina Perrone, Assunta Melaccio, Pierluigi De Santis, Anna Natalizia Santoro, Chiara Guarini, Maria Laura Iaia, Palma Fedele

**Affiliations:** 1 Medical Oncology, Dario Camberlingo Hospital, 72021 Francavilla Fontana, Italy; carmelo.laface@asl.brindisi.it (C.L.); martina.perrone@asl.brindisi.it (M.P.); pierluigi.desantis@asl.brindisi.it (P.D.S.); annanatalizia.santoro@asl.brindisi.it (A.N.S.); chiara.guarini@asl.brindisi.it (C.G.); marialaura.iaia@asl.brindisi.it (M.L.I.); palma.fedele@asl.brindisi.it (P.F.); 2 Medical Oncology, San Paolo Hospital, ASL Bari, 70123 Bari, Italy; francesco.giuliani@asl.bari.it (F.G.); assunta.melaccio@asl.bari.it (A.M.)

**Keywords:** prostate cancer, immunotherapy

## Abstract

Prostate cancer (PC) is the most common type of tumor in men. In the early stage of the disease, it is sensitive to androgen deprivation therapy. In patients with metastatic castration-sensitive prostate cancer (mHSPC), chemotherapy and second-generation androgen receptor therapy have led to increased survival. However, despite advances in the management of mHSPC, castration resistance is unavoidable and many patients develop metastatic castration-resistant disease (mCRPC). In the past few decades, immunotherapy has dramatically changed the oncology landscape and has increased the survival rate of many types of cancer. However, immunotherapy in prostate cancer has not yet given the revolutionary results it has in other types of tumors. Research into new treatments is very important for patients with mCRPC because of its poor prognosis. In this review, we focus on the reasons for the apparent intrinsic resistance of prostate cancer to immunotherapy, the possibilities for overcoming this resistance, and the clinical evidence and new therapeutic perspectives regarding immunotherapy in prostate cancer with a look toward the future.

## 1. Introduction

Prostate cancer (PCa) is one of the malignancies that affects middle-aged men between the ages of 45 and 60 and contributes significantly to the increase in mortality rates in men globally [1]. PCa-related risk factors include family risk, ethnicity, obesity, and age. PCa tumorigenesis is dependent on epidemiology and genetics [1]. Chromosomal rearrangements have an important role in the development of cancer and, in this context, the role of genetic alterations on androgen synthesis and metabolism has already been investigated as PCA tumorigenesis is closely related to the androgen receptor signaling pathway. Many men with PCa are diagnosed through prostate-specific antigen (PSA) testing, digital rectal examination, prostate biopsy and analysis, and magnetic resonance imaging [2,3]. PCa can be classified as androgen-sensitive (HSPC) or castration-resistant (CRPC), based on the sensitivity to androgen deprivation therapy [4]. There are several treatment options for patients with PCa that can be used alone or in combination with each other; these are chosen based on stage, potential side effects, and patient choice, and include surgery, radiation therapy, cryotherapy, active surveillance, hormone therapy, and chemotherapy [5,6,7,8,9,10,11]. However, each treatment should be chosen taking into account the side effects, which can also be serious, such as incontinence, erectile dysfunction, hematological toxicity, asthenia, and peripheral neuropathy. Metastasis and the eventual development of resistance can occur during the initial treatment [6,7,8,9,10,11,12]. For all of these reasons, the introduction of new well-tolerated and effective treatment strategies is necessary. Immunotherapy has changed the natural history of several cancers and revolutionized modern oncology. It generally consists of the stimulation of the immune system against tumor cells [13]. It has also been investigated in Pca, both with drugs against the PD1-PDL1 axis, CTLA, and with the use of vaccines and adoptive cell immunotherapy. PCa is traditionally considered an immunologically “cold” tumor with low expression of PD-L1 and an immunosuppressive tumor microenvironment (TME). In this review, we aim to provide a holistic overview of immunotherapy in PCa, including a description of the molecular mechanisms, the studies in progress, and future treatment options.

## 2. PCa as a Cold Tumor: Mechanisms of Immune Tolerance

**Figure 1:**
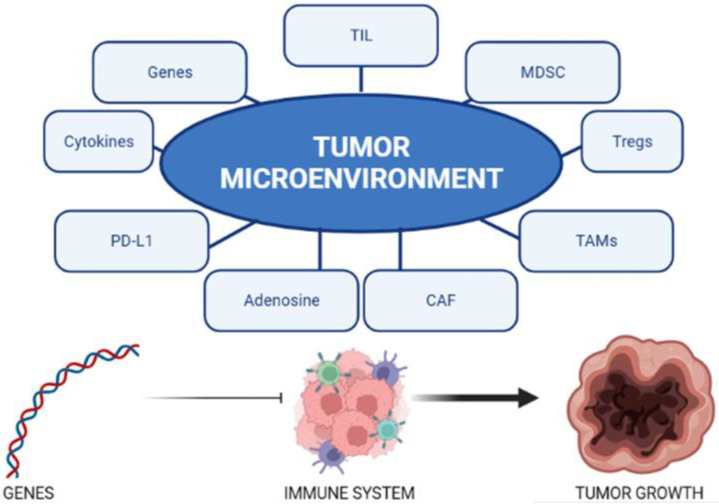
The tumor microenvironment is influenced by multiple factors. The genetics of PCA influence the interaction between the immune system and the tumor.

PCa is traditionally regarded as a cold tumor. This is due to the characteristics of the tumor microenvironment and its genetic expression. The main mechanisms responsible for the intrinsic resistance of PCa to immunotherapy are listed below and are shown in Figure 1.

### 2.1. Tumor Microenvironment (TME)

The TME consists of tumor cells, the surrounding blood vessels, an extracellular matrix, immune cells, and signaling molecules that regulate growth and communication between the tumor cells and their surroundings [14]. The composition of the inflammatory microenvironment is crucial in the antitumor immune response, and vascularization has a role both in causing hypoxia (which causes an immunosuppressive environment) and in changing the metabolic state of the TME [15,16]. The TME in PCa tends to be non-inflammatory and with a low expression of neoantigens, which are crucial in the stimulation of the immune system.

Tumor immune evasion is highly correlated with the upregulation of B7 inhibitory molecules in the TME. CD276 is a member of the B7 family that is involved in tumorigenesis. It has stimulating roles, as it is involved in the activation of T cells and the production of interferon-gamma (IFN-γ), but it is also suppressive in immune responses because it promotes metastasis [17].

CD276 is correlated with the reduced function of TMJ and BRCA, as well as low tumor-infiltrating lymphocytes (TIL), and it is highly expressed in advanced Pca [18,19,20].

The elements involved in the composition of the anti-inflammatory TME in PCa are listed below.

#### 2.1.1. Tumor-Infiltrating Lymphocytes (TIL)

The presence of TIL is related to a better prognosis [16]. However, PCa’s TIL populations are composed primarily of M2-type tumor-associated macrophage cells (TAM) and of CD4+ FOXP3+ CD25+ Treg cells, which contribute to the production of immunosuppressive cytokines [18,19,20]. Effector T cells are divided into two types: Th1 and Th2. TILs increase during androgen-deprivation therapy in AR-dependent tumors and, in addition, a reduction in Treg cells occurs [21]. The upregulation of immune checkpoints and TILs has been observed in tissues from patients receiving ADT therapy. TILs can be used as a therapy that involves the use of specific lymphocytes around the tumor. T cells against malignant tumor cells are extracted and they are stimulated to proliferate around the tumor. This therapy is highly effective in patients with metastatic melanoma, with response rates of approximately 40% and complete response rates of 10% to 20% [22]. Furthermore, it has also demonstrated efficacy in preclinical studies in patients with lung cancer [23]. TIL therapy is divided into several phases, starting with surgically resected tumor tissue, from which lymphocytes are extracted and expanded in an interleukin (IL)-2 medium for 4 weeks. Subsequently, the TILs are expanded for 14 days with a procedure that allows their rapid expansion with the anti-CD3 antibody. The TILs are then prepared for infusion.

It is difficult to effectively integrate TIL-based immunotherapy into prostate cancer treatments for several reasons, including the immunosuppressive PCa microenvironment and high tumor heterogeneity. Prostate cancer has a relatively low mutation rate (~0.9 mutations/Mb); however, in advanced stages, alterations in the DNA damage repair mechanisms increase the mutational burden in patients. In several studies, adoptive immunotherapy with direct T cells has demonstrated efficacy, even in tumors with a lower mutational burden [24]. Experiments on TILs in prostate cancer have recently been performed and they indicate a feasibility of tumor-responsive TIL extraction within prostate cancer. In an in vitro study, cultures of prostate-TIL were extracted and proliferated with an expansion rate of approximately 50%. An expression of chemokine receptors was present after expansion. However, proliferating CAR and TIL T cells in immunosuppressive environments for long periods of time remains a major challenge and, in this regard, the goal may be to increase the CAR T cell survival rates. This could occur through the incorporation of TIL 4-1BB and CD137 receptors [25,26].

#### 2.1.2. Myeloid-Derived Suppressor Cells (MDSC)

MDSCs are a diverse group of immune cells of the myeloid lineage [27]. MDSCs are hematopoietic cells that suppress the antitumor activity of the immune system, and when present in TME, are associated with a poor prognosis in several tumor subtypes [28,29,30,31]. The role of MDSC in Pca’s immune evasion is very important [32]. Their immunosuppressive properties include the induction of NK cell anergy, the inhibition of T cell activation, the promotion of the de novo expansion of Tregs, and the inhibition of the maturation of dendritic cells [33]. Some data have shown that the cytokine IL23 secreted by MDSCs can promote cell proliferation by activating AR signaling in PCa cells, and the IL23 blockade restores sensitivity to androgen therapy [34,35,36,37]. MDSCs have been found in the peripheral blood of patients with PCa compared to age-matched healthy controls and the MDSC level was associated with disease burden and PSA level [38]. MDSCs represent a major barrier to many cancer immunotherapies, and targeting them may be an advantageous strategy to improve the efficiency of immunotherapy [38].

#### 2.1.3. Treg Cells

T-cell regulators/T-cell suppressors (Tregs) have the main role of stopping the T-cell-mediated immune response, and thereby promoting immune tolerance [39]. The CD80 and CD86 present on dendritic cells (DC) bind to the CTLA-4 expressed on Tregs, and in this way, the activity of T cells is inhibited [40,41]. Through galectin-1, they can prevent the proliferation of T cells by increasing apoptosis and also produce immunosuppressive cytokines (TGF-β and IL-10) [40,41,42]. In the PCa’s TME, Tregs support MDSCs and M2 macrophages downregulating cytotoxic lymphocytes and NK cells (CTLs) [43]. A histone deacetylase inhibitor, called Entinostat, activates STAT3 acetylation and reduces FoxP3 expression, which inhibits the proliferation and growth of Treg populations, suggesting a novel approach to modulating the TME in PCa [44].

#### 2.1.4. Tumor-Associated Macrophages (TAMs)

TAMs are a key component of the inflammatory TME [45]. In general, they can be classified into an M1 anticancer phenotype or an M2 protumor phenotype. However, mixed phenotypes have been observed. TAMs can stimulate the genetic instability of cancer cells and, consequently, their proliferation and migration [46,47]. In PCa, higher TAM density has been associated with shorter cancer-specific survival and a higher Gleason score [48,49,50]. In addition, they increase the activation of osteoclast-related pathways and have been associated with the activity of TAM, and this is very interesting given the particular tropism of the PCa for bone [48,49,50,51].

#### 2.1.5. Stromal Cells and Cancer-Associated Fibroblasts (CAF)

Various cell types take part in the TME, each with a different task, such as: mesenchymal stromal cells (MSCs), pericytes, fibroblasts, endothelial cells, pericytes, and other innate and adaptive immune cells [52]. CAFs and endothelial cells promote extracellular matrix remodeling (leading to tumor cell invasion), growth factor expression, and neo-angiogenesis [52,53,54]. Among the most important growth factors are bFGF, PDGF, and TNF-α. In addition, MMP and VEGF promote a more aggressive drug-resistant PCa phenotype [53,55]. CAFs perform their antitumor activity on cytotoxic lymphocytes with the development of hypoxia and local inflammation and, therefore, the infiltration of immunosuppressive MDSCs and Tregs in the TME [52,53,56], the overexpression of CD73 and CD39—which generates immunosuppressive adenine, the release of lactate, the expression of PD-L1 mediated by IL-1, and the activation of TGF-β signaling, which involves resistance to therapy with anti PD1 [56,57,58].

#### 2.1.6. Adenosine in PCa

Adenosine is a small anti-inflammatory mediator that suppresses the cytotoxic function of TILs and can increase Tregs, MDSC, fibroblasts, and the function of dendritic cells [59,60]. Adenosine is highly expressed in PCa and causes immune evasion. It is produced by dephosphorylation from ATP that is catalyzed by ectonucleotidases, such as prostatic acid phosphatase (PAP), CD39, CD73, and alkaline phosphatase, and it is associated with immunosuppressive activity, while the hazard-associated molecular pattern (DAMP) promotes immune responses [61,62,63,64,65].

However, studies of vaccination against PAP have been quite disappointing. Early clinical trials are now investigating the efficacy of A2UN and/or A2B receptor antagonists and inhibitors of CD73 ectonucleotidase in PCa [64,65,66,67,68].

#### 2.1.7. Expression of Programmed Death Ligand-1 (PD-L1)

PD-L1 is a protein expressed at low levels in different cell types, such as T lymphocytes, epithelial cells, macrophage endothelial cells, and dendritic cells; it is part of the B7 protein family and binds the PD-1 receptor. The expression of PD-L1 has been detected in only 12% of patients with MSI-H/dMMR disease.

In approximately 10% of tumors with MSI-H/dMMR deletion of PTEN, there is an increased PD-L1 expression. PD-L1 is less expressed in PCa than in other tumors and it also varies according to the histological subtype (it was detected in 46% of neuroendocrine PCa, 29% of acinar Pca, and 7% of ductal PCa) [69].

In addition, the expression level of PD-L1 may be related to the Gleason score, metastasis, infiltration, and biochemical recurrence; however, the data in this regard are not unique [70,71,72,73,74].

#### 2.1.8. Androgen Receptor Signaling

Androgen receptor (AR) signaling is involved in the growth of the survival and tumorigenesis of prostate cells. Deprivative androgen therapy (ADT) that reduces testosterone and AR signaling may be a therapeutic opportunity [75].

The effect of ADT on antitumor immune response and tolerance is controversial [76,77]. In castration-sensitive tumors, ADT leads to a reduction in Tregs and an increase in TIL, while in patients with castration-resistance disease, there is a simultaneous increase in immune tolerance [78].

#### 2.1.9. Tumoral Cytokines

Cytokines are involved in the immune response [79] and participate in the progression of PCa [80]. Pro-inflammatory cytokines lead to increased immune cell infiltration of both cytotoxic T lymphocytes and immunosuppressive MDSCs and Tregs (mainly due to IL-1β and IL-2 production) [81,82]. Many anti-inflammatory cytokines participate in PCA tumorigenesis, such as IL-4, IL-6, and IL-10, which have been found to be elevated in the serum of CRPC patients (82). Tumor necrosis factor α (TNF-α) and IL-17 increase the expression of PD-L1, which has an immunosuppressive role [82]. Furthermore, TGF-β and IL-10 are associated with a weak autoimmune response [83,84].

### 2.2. Genes Involved in the Regulation of the Immune System in PCa

#### 2.2.1. PTEN

PTEN is a tumor suppressor gene that plays an important role in carcinogenesis and metastasis as it regulates migration and infiltration. About 10% of PCa patients have a homozygous loss of PTEN and 50% have a heterozygous loss [85,86]. In addition to its function on PCa biology, PTEN affects the composition of the inflammatory microenvironment of the TME extension [87,88].

PTEN deficiency leads to increased MDSC infiltration and expression of immunosuppressive molecules IDO1 and CD276 and several chemokines (CXCL12 and CXCL8). It is also related to a high Gleason’s score on ADT sensitivity [87,88,89]. Developing novel PI3K-AKT-mTOR inhibitors and reactivating PTEN function could be a novel therapeutic strategy in CRPC patients with PTEN loss. In a preclinical study, mice genetically modified for the absence of PTEN/p53 were subjected to ADT (degarelix), an anti-PD-1 antibody, or a PI3K inhibitor (copanlisib) in monotherapy or in combination therapy. TAMs with PD-1 expression counteract the antitumor action of ADT/PI3Ki. The combination of ADT/PI3Ki with anti-PD-1 induced an increase in TAM-dependent anti-tumor responses. Furthermore, the tumor cells undergoing PI3Ki had a decrease in lactate production and, consequently, histone lactylation within the TAM. This resulted in the phagocytic activation of TAMs against tumor cells, further stimulated by anti-PD1 and ADT therapy [90].

#### 2.2.2. Forkhead-Box A1 (FOXA1)

FOXA1 has a regulatory function on chromatin and can also influence the TME. FOXA1 causes immunosuppression and promotes the infiltration of M2-type macrophages [91].

Moreover, it was discovered that, in PCa patients with low expression of FOXA1, there is an upregulation of the genes that cause the inflammatory response [91].

FOXA1 overexpression in patients with PCa is inversely correlated with NFI signaling activity and with the expression of the genes that regulate the presentation of antigens, contributing to the resistance to immune checkpoint inhibitors [92,93].

#### 2.2.3. EZH2

EZH2 is a key mediator related to an immunosuppressive TME, the inhibition of T cell differentiation and infiltration, and resistance to immunotherapy. It also regulates the expression of the major histocompatibility complex (MHC) and is involved in Treg and CD4+T differentiation. Furthermore, EZH2 negatively rules INF-induced genes [94,95].

#### 2.2.4. Dickkopf-1 (DKK-1)

DKK-1 is a member of the Dickkopf bone factor family and promotes angiogenesis through VEGFR2. Elevated levels of DKK-1 are associated with a poor prognosis in various tumors. This could be due to its immunosuppressive effect [96,97].

The increase in DKK-1 expression in PCa may have direct effects on the cell cycle, thus increasing tumor proliferation. Furthermore, high serum DKK-1 levels dosed at the time of diagnosis have been correlated with a worse prognosis. Analysis of PCa immune cells revealed that the levels of DKK1 led to an increase in M2 macrophages and lower levels of activated NK and CD8+ T cells [98,99].

In human models of PCa (PC3), DKK1 inhibition blocked tumor growth through the activation of NK cells [98,99,100,101].

A parallel arm Phase 1b/IIa study of DKN-01, a DKK1 inhibitor, as a monotherapy or in combination with docetaxel in patients with advanced PCa and elevated DKK-1 levels is underway (NCT03837353).

#### 2.2.5. Wolf-Hirschhorn Syndrome Candidate Protein 1 (WHSC1, Also Known as MMSET and NSD2)

WHSC1 is correlated with an immunosuppressive TME and has a role in tumor progression in PCa as it mimics lymphocyte infiltration and reduces antigen presentation [102]. The pharmacological inhibition of WHSC1 in a mouse model of PCa (TRAMP C-2) upregulated MHC-II expression on CD45+CD11c+ Dcs and reduced CD4+CD25+ Treg infiltration. It also alters DNA methylation and chromatin accessibility in order to interact with the ubiquitinase and proteasome genes, and it is involved in the expression of CD276 and PD-L1 [103,104,105].

#### 2.2.6. NKG2D

NKG2D is an activating receptor that has a role in killing cancer and virus-infected cells and is expressed on the surface of NK, CD56+, and CD8+ T cells. In patients with Pca, there is a dysfunction of NK cells expressing activating receptors and inhibitors on their surfaces, which leads to poor pharmacological results [106,107]. The blood levels of the NK activating receptor (NKp46) were decreased in patients with PCa and they relate in an inversely proportional way with PSA levels [108,109]. PCa cells, through exosomes containing the surface ligand MICA/B and ULBP-2, negatively regulate the cytotoxic function of NK cells, reducing the expression of NKG2D [110]. One promising approach could be restoring NK cell activation.

#### 2.2.7. CD38

CD38 is part of the ADP-ribosylcyclase family and has an important role in the immune evasion of tumors. In fact, it alters the TIL function by depleting NAD+; on the contrary, the inhibition of CD38 causes the metabolic reorganization of T cells [111,112,113]. Targeted therapies against this CD38/NAD+ axis may make anticancer T-cell adoptive therapy more effective. The combination of anti-PD-1 (cemiplimab) and anti-CD38 (isatuximab) monoclonal antibodies resulted in the activation of peripheral T cells in patients with mCRPC and a median reduction in CD38+ tumor-infiltrating immune cells, from 40% to 3% (NCT03367819) [114].

#### 2.2.8. Polycomb Repressive Complex 1 (PRC1)

The repressive complex Polycomb 1 (PRC1) plays a role in transcriptional regulation. PRC1 promotes PCa immunosuppression through the recruitment of MDSC, TAM, and Tregs in the TME. In contrast, PRC1 inhibition had an antiangiogenic effect and reduced bone metastasis in a DNPC model (Ptenpc−/−Smad4pc−/−) [115].

#### 2.2.9. PIKfyve

PIKFYVE belongs to a family of lipid kinases that are related to immune checkpoint inhibition. The inhibition of PIKfyve blocks Toll-like receptors (TLRs) and the IL12/IL13 signaling pathways inhibit the immune response [116]. Using the drug ESK981, a multi-tyrosine kinase inhibitor that primarily targets VEGFR-1 and 2, the activity of PIKfyve has been inhibited.

Combined treatments of PIKfyve knockdown with anti-PD-1 therapy have been investigated and, in preclinical studies, this led to a significant increase in tumor response [117]. Phase II clinical trials are investigating the efficacy of ESK981 in combination with nivolumab (NCT04159896) or as a monotherapy (NCT03456804) [117].

## 3. Immunotherapy in PCa: Studies Evaluating Single Agent Immune Checkpoint Inhibitors (ICI) in mCRPC and mHSPC

Immune checkpoints function as controllers of immune activation, and are crucial for preserving immune balance and averting autoimmune reactions. In cancer, tumor cells frequently trigger these checkpoint processes to inhibit the immune system’s anti-cancer responses. The development of checkpoint inhibitory antibodies has led to a significant transformation in the treatment of various hematologic and solid malignancies, with unprecedented response rates reported [118,119].

However, not all solid tumors have reported equally encouraging results. The emphasis on immune checkpoint inhibitors (ICI) has also led to many trials in PCa, particularly in metastatic disease, but unfortunately, the results have been disappointing thus far. The role of the tumor microenvironment is key to understanding the functioning of ICI; it needs to be investigated qualitatively rather than quantitatively, given the genomic determinants of PCa cells. PD-L1, TMB, or dMMR/MSI-high can be considered biomarkers of immunotherapy, and new biomarkers are being added, such as specific pathway aberrations (e.g., AR-V7, HRD, CDK12 inactivated tumors) [120].

Phase I and II studies have explored antibodies targeting the programmed death receptor 1 (PD-1) and T-cell antigen-4 (CTLA-4) inhibitors in metastatic castration-resistant prostate cancer (mCRPC); however, the results obtained were not brilliant [121]. A 2007 study was conducted in patients with hormone refractory prostate cancer; the objective was to evaluate the efficacy of a single 3 mg/kg dose of the humanized anti-CTLA-4 IgG monoclonal antibody, ipilimumab. The endpoints were serologic measures of autoimmunity and the evaluation of T-cell activation, and the treatment with ipilimumab resulted in polyclonal T-cell activation. Unfortunately, there was no objective response, but benefits in the biochemical response were observed. Immune adverse events were limited to one patient with grade 3 rash and pruritus [122]. A randomized phase III study conducted in 2017 (CA184-095) used high-dose (10 mg/kg) ipilimumab monotherapy and had OS as its primary endpoint OS, and PFS and safety as the secondary endpoints. This study did not detect an advantage in the median OS over the placebo (28.7 months vs. 29.7 months; HR = 1.11, 95% CI 26.1–34.2 months, *p* = 0.3667) in chemotherapy-naïve patients with minimally symptomatic mCRPC [123]. Conversely, ipilimumab dose escalation lengthened the median PFS (5.6 months vs. 3.8 months; HR = 0.67, 95.87% CI 0.55–0.81) and decreased the PSA levels (23% vs. 8%); however, unfortunately, the incidence of grade 3–4 adverse reactions and treatment-related deaths also increased.

Similar outcomes were seen in a phase III study (CA184-043) in which patients with CRPC bone metastases progressing after docetaxel therapy were randomized to receive bone-targeted radiotherapy, followed by ipilimumab 10 mg/kg or a placebo [124].

Countless studies have also investigated anti-PDL-1 monoclonal antibodies in mCRPC. Among the best known, the phase Ib study KEYNOTE-028 considered a cohort of 23 pretreated mCRPC patients with measurable disease and PD-L1 expression ≥1% in tumor or stromal cells. In this study, the primary endpoint was ORR, and the secondary endpoints were OS, PFS, and DOR. The anti-PDL-1 pembrolizumab generated an objective response rate (ORR) of 17.4%, with four patients exhibiting a partial response and three of the four patients exhibiting a biochemical response. As in similar studies, the monoclonal antibody was very well tolerated, prompting further investigation [125].

The KEYNOTE-199 study evaluated the efficacy of pembrolizumab monotherapy in three patient cohorts: PD-L1 positive tumor and measurable disease; PD-L1 negative tumors and measurable disease; and non-measurable metastatic bone disease regardless of PD-L1 status. The primary endpoint of the study was ORR and the secondary endpoints were safety, DCR, DOR, PSA response rate, PFS, OS, and duration of PSA response. The median overall survival and objective response rates were modest, but pembrolizumab demonstrated activity in both RECIST measurable diseases and bone-predominant diseases, regardless of PD-L1 expression.

The DNA damage repair (DDR) gene mutation status, as determined by whole-exome sequencing and analysis, has not revealed a clear relationship between pembrolizumab response and DDR gene mutations [126]. DDR gene alterations, found in 23% of mCRPC patients, mainly involve the BRCA2, ATM, CHEK2, and BRCA1 genes. These alterations generate genomic instability and reactivity of the antitumor immune response by increasing the mutational load of the DNA [127,128,129]. Atezolizumab (anti-PDL-1) has been used (schedule 1200 mg IV every three weeks) in patients with mCRPC previously treated with enzalutamide and/or sipuleucel-T, demonstrating a good safety profile, an overall survival rate of twelve months survival of 55.6%, and a six-month progression-free survival rate of 26.7%. The median overall survival is still under definition [130]. The JAVELIN solid tumor study tested Avelumab, another human IgG1 monoclonal antibody that binds to PD-L1, at different doses administered every 2 weeks [131]. Among the various dosages, the 10 mg/kg dose was chosen as the most effective; this was evaluated in an expansion cohort of 18 mCRPC patients who had progressed on previous treatments. Avelumab had a manageable safety profile. The results were not impressive: seven patients evaluated by RECISTv1.1 achieved stable disease after 24 weeks of treatment and six patients experienced disease progression at 6 weeks. The prolonged PSA doubling time (PSADT) was stable or declining [132].

However, while encouraging in the preclinical stages, the activity of the immune checkpoint blockade in prostate cancer is not convincing, especially with ICI monotherapies in real-world experiences, compared to that observed in other cancers. There are several biological causes for this failure. From an immunological point of view, PCa has a much lower tumor mutation burden (TMB) than other solid tumors that are considered “immunogenic”. In addition, we must consider the lower PD-L1 expression levels compared to other cancers.

The tumor microenvironment is made hostile to the survival of T cells and TIL through various mechanisms due to hypoxic zones within the PCa. It is further made immunosuppressive through the prevalence of myeloid-derived suppressor cells (MDSCs) and tumor-associated macrophages, at the expense of immature myeloid cells. CD4+ FOXP3+ CD25+ and CD8+ FOXP3+ CD25+ T cells release inhibitory cytokines, and the IFN-1 pathway associated with PTEN gene loss directs the microenvironment to suppress the immune response. These conditions result in a poor response to the immune checkpoint blockade [133]. All of the studies evaluating single agent immune checkpoint inhibitors (ICI) in mCRPC and mHSPC are summarized in Table 1.

## 4. Vaccine-Based Immunotherapy

Sipuleucel-T is a vaccine that contains autologous peripheral blood mononuclear cells (PBMCs) that are reinfused in patients after in vitro culture. They are prepared in vitro with a recombinant fusion protein (PA2024) containing prostatic acid phosphatase, prostate antigen, and granulocyte-macrophage colony stimulating factor (GM-CSF) by activating APCs to expand the antigen [134,135]. A double-blind phase III, randomized, multicenter study (IMPACT: NCT00065442) of 512 patients in a 2:1 ratio received sipuleucel-T or a placebo with overall survival as the primary endpoint. A relative reduction in the risk of death of 22% was observed in the experimental group compared to the placebo group, with a 4.1 month increase in median survival (25.8 months vs. 21.7 months, respectively, in the experimental group vs. placebo). The 36-month survival probability was 31.7% vs. 23.0% in the sipuleucel-T vs. placebo group, respectively. Regarding safety, the most frequent adverse events in the sipuleucel-T group were headache and fever [134]. Studies on the combination of this vaccine and target agents AR [136], atezolizumab [137], or radio-223 [138] in patients with mCRPC are ongoing. It is the only vaccine against PCa approved by the FDA.

GVAX is another vaccine and is based on genetically modified PCa cells that produce the granulocyte-macrophage colony-stimulating factor [139]. GVAX is a safe cytokine and causes an immune response in a dose-dependent manner. The patients showed only fever and flu-like symptoms during treatment. However, considering several failed phase III studies of this vaccination, further trials have been abandoned [140,141].

PROSTVAC is a vaccine that uses a recombinant vaccine strain coupled with transgenes and costimulatory molecules to elicit an immune response [142]. Patients who were treated with PROSTVAC showed increased levels of specific PSA T cells [142]. In a phase II study, 125 patients with mCRPC and a Gleason score ≤7 were randomized to receive PROSTVAC or a placebo [143], with a median OS of 24.4 months in the investigational arm and 16.3 months in the placebo arm [143]. A recent phase III study of PROSTVAC was conducted, but it is considered negative [144,145].

DNA vaccines have been tested in animals but their use in humans remains controversial because of the high risk–benefit ratio [135]. They offer a novel approach [146] compared to other cancer vaccinations in terms of the absence of infectious agents. Several phase I clinical trials are currently ongoing [147,148].

## 5. Adoptive Immune Cell Immunotherapy

Adoptive cell therapy is based on the use of autologous immune effector cells activated to attack the cancer cells and it has obtained promising results in a variety of hematologic malignancies [149]. However, T cell activity is reduced due to the immunosuppressive environment of the TME caused by the expression of inhibitory molecules (such as PD-1) by cancer cells. The epithelial cell adhesion molecule (EpCAM) is effective in a broad range of cancer immunotherapies involving this stem cell antigen [150]. To more specifically target PCa, CAR-T cells contain the prostate-specific membrane antigen (PSMA). In an initial phase I study of PSMA targeting TGFβ-insensitive armored CAR T cells (NCT0308-9203) in patients with CRPC, 5 of 13 patients developed grade 2 or higher cytokine release (CRS) and 4 had ≥30% reductions in PSA. One patient achieved a >98% reduction in PSA. However, this patient developed enterococcal sepsis 30 days after infusion, leading to multiorgan dysfunction and death [151].

Recent experiments on TILs in PCa have indicated the potential to expand TILs that are reactive against PCa. In one study, 28 cultures of TILs resulting from PCa were successfully extracted and expanded in vitro. The analysis revealed the expression of chemokine receptors after expansion [25,152]. The results of these studies are promising, but CAR-T cell therapy and TILs still face many challenges because the TME in PCa is immunosuppressive and it limits the efficacy of adoptive immunotherapy.

Bispecific T-cell engagement (BiTE) antibodies target PSMA and use T-cells via the CD3 receptor. AMG 212 (pasotuxizumab) has been studied in a phase I clinical trial in 68 patients after disease progression from abiraterone or enzalutamide and at least one taxane-based chemotherapy. There have been dose-dependent reductions in PSA and objective answers in one-third of patients [153]. The limitation of this study included the development of drug-neutralizing antibodies with subcutaneous injection and the short serum half-life of the molecule. In this regard, there is an ongoing development of BiTE molecules with an extended half-life [154]. Other targets for BiT therapy that are expressed in different PCa subtypes are being studied, including delta-like protein 3 (DLL3), human carcinoembryonic antigen (CEA)-related cell adhesion molecule 5, and six-transmembrane prostate epithelial antigen (STEAP) [155,156].

## 6. Immunotherapy Combination Strategies

Considering the poor results of immunotherapy as single agents, immune checkpoint inhibitors have been studied in combination with each other or with other drugs. These studies aim to overcome the inherent resistance of PCa to immunotherapy by stimulating the immune system in a combined way and improving cancer immunotherapy. Combination therapies with multiple strategies or drug combinations targeting specific mechanisms in the TME aim to overcome the resistance mechanisms in PCa.

### 6.1. Immunocheckpoint Inhibitors in Combination with ARSI

Considering the role of the AR blockade on the TME, in a phase I study in mHSPC patients with recurrent non-metastatic PCa, tremelimumab (a humanized anti-CTLA-4 antibody) was administered in combination with bicalutamide. The primary endpoint of the study was safety and the secondary was measures of PSA kinetics and the identification of a maximum tolerated dose. Three of the eleven patients experienced a stretch of double-time PSA [157].

AR is a negative regulator of CD8+ T cells in patients with mCRPC; thus, it decreases the response to anti-PD1/PD-L1 treatment [76,77].

A phase II single-arm study in 28 patients with mCRPC progressing after enzalutamide investigated the combination of pembrolizumab with enzalutamide. The primary outcome was a prostate-specific antigen (PSA) decline of ≥50% and the secondary endpoints included ORR, PSA-PFS, OS, and time to subsequent treatment. The PSA response rate was 18% (5/28) and the OS was 21.9 months in all patients. In the responder subgroup (three patients), the OS was 41.7 months [158], one was MSI-high, and none had detectable PD-L1 expression in biopsy tissues [158].

In the randomized phase III IMbassador 250 (#NCT03016312) study in 759 patients progressing after abiraterone or docetaxel, enzalutamide was studied in combination with atezolizumab or as a monotherapy. The primary endpoint (OS) was not met [159]. However, the results are interesting as they suggest that the combination of atezolizumab plus enzalutamide may be more effective in patients with high levels of CD8+ T cells and high PDL1 expression [159].

KEYNOTE-641, a multicenter, randomized, double-blind, phase III study is currently underway in approximately 1200 patients with mCRPC to evaluate the efficacy and safety of enzalutamide plus pembrolizumab+ versus enzalutamide plus placebo (NCT03834493). The primary outcomes of this study are OS and rPFS, and the results have not been published [160].

Further ongoing studies are summarized in Table 2.

### 6.2. Immunocheckpoint Inhibitors in Combination with Cryotherapy or Radiotherapy

ICIs have not been successful in the early stages of the hormone-sensitive disease. Ross and colleagues conducted a single-institution, single-arm pilot study in patients with oligometastatic HSPC to evaluate pembrolizumab in combination with prostate cryotherapy and ADT. Exploiting the increased response to ICI caused by local ablative treatment, the study allowed good local control of the disease with a significant reduction in PSA in 42% of the 12 enrolled patients [161].

The other ongoing phase Ib to phase III studies in mHSPC have evaluated the combination of ICIs with other drugs. Several data have also confirmed the role of radiotherapy in the production of neoantigens. These could stimulate the immune response. Based on this rationale, a Phase I/II study in 50 patients with mCRPC evaluated the treatment with ipilimumab alone or in combination with radiation therapy. One of them had a complete answer, eight had a PSA drop of ≥50%, and six had stable disease. These responding patients had increased intratumoral T-cell infiltration [162].

A phase II study in 31 men with mCRPC previously treated with anti-androgenic agents of avelumab with stereotactic radiotherapy showed a median OS of 14.1 months [163].

In a double-blind, phase III study, patients with mCRPC were randomized to receive radiotherapy followed by ipilimumab or a placebo. There was no statistically significant difference in the OS, but only an increase in progression-free survival between the ipilimumab group and the placebo group. However, in the long-term follow-up study, the OS in the ipilimumab arm was 7.9% vs. 2.7% in the 5-year placebo arm [164].

Further ongoing studies are summarized in Table 2.

### 6.3. Immunocheckpoint Inhibitors in Combination with Chemotherapy

The association of chemotherapy with immunotherapy has also been investigated to overcome the resistance of PCa to immunotherapy. The advantage of this association consists of the fact that the cytotoxic effect of chemotherapy changes the molecular arrangement of tumor cells, thus facilitating the action of immunotherapy.

KEYNOTE-365 (NCT-02861573), a phase 1b/2 study in patients with mCRPC not pretreated with abiraterone or enzalutamide and not with chemotherapy, investigated the combination of docetaxel and prednisone plus pembrolizumab and demonstrated an OS of 20.2 months, an ORR of 23%, a median radiographic PFS (rPFS) of 8.5 months, and a PSA response rate of 34% [165].

KEYNOTE-921, a multicenter, randomized, double-blind phase III study in this patient population is ongoing to evaluate docetaxel and prednisone or prednisolone plus pembrolizumab versus docetaxel plus a placebo with the primary endpoints of OS and rPFS [166]. Further ongoing studies are summarized in Table 2.

### 6.4. PARP- Inhibitors and Immunocheckpoint Inhibitors

Most CRPC patients with DDR gene alterations have unsatisfactory responses to immunotherapy and it is not clear whether there is a correlation between the PD-L1 and BRCA status [167].

PARP inhibitors can induce replication stress that causes the entrance into the cytosol of genetic material and the formation of highly permeable micronuclei, even in HR-proficient cells [168,169,170]. This causes a potent immune response because of the transcription of a Type I Interferon. This is one of the possible mechanisms of the synergic efficacy of PARP inhibitors and immunotherapy. PARP inhibitors also upregulate immune checkpoints such as the programmed death-ligand 1 (PD-L1) [171]. The combination of PARP inhibitors and immune checkpoint inhibitors can increase PD-L1 expression in cells with BRCA2 mutation and allows a higher release of neoantigens and consequent increases in T-cell responses against tumors [172,173]. PARP/PI3K inhibition causes the release of DNA-DSB-containing microvesicles that activate the macrophages with an anti-tumor effect in mouse models; this suggests that PARP/PI3K inhibition can augment the response of ICI [174,175].

Clinical studies have investigated the combination of PARP inhibitors and PD1 and PD-L1 blockers in patients with mCRPC. Rucaparib plus nivolumab has been investigated in patients with mCRPC who progressed to at least one line of NHT in a phase I study (NCT03572478) [176]. PSA response to treatment was observed in only one patient (who has a pathogenic BRCA2 mutation). The KEYNOTE-365 trial (NCT0286-1573) was a phase 1–2 trial conducted in post-docetaxel patients with no detectable HR mutation who progressed after at least two lines of NHT. The study had ten cohorts and evaluated pembrolizumab in combination with other drugs. The primary endpoint was the percentage of participants with a decrease of ≥50% in PSA, Aes, and ORR. The cohort A patients received pembrolizumab + olaparib, and this combination of agents showed activity in docetaxel-pretreated patients [177,178]. A phase I/II study showed the efficacy of the combination of olaparib with durvalumab in mCRPC patients. The median radiographic PFS was greater in patients with DDR defects than in patients without DDR defects (16.1 months vs. 4.8 months) [179] and were found to be correlated to a higher amount of Ki-67+/PD-1+/CD8+ T-cells, a decrease in circulating tumor cells after treatment, and an increase in dendritic cells [179]. The combination of olaparib and durvalumab is being studied in a phase II clinical trial in patients with biallelic CDK12 mutations (Cohort A), mismatch repair deficiency (Cohort B), or HRR gene mutations (Cohort C), and the primary outcome is undetectable PSA. Patients in cohorts A and B will receive three cycles of durvalumab followed by three cycles of olaparib in combination with durvalumab; cohort C will receive six cycles of durvalumab plus olaparib [180]. A three-arm non-randomized phase II study in patients with mCRPC, Checkmate 9KD (NCT03338790), examined the combination of nivolumab with rucaparib. The study shows greater objective responses for HRD+ vs. HRD– tumors, and in mutated BRCA2, the ORR was 37.5% [181]. Different drugs kill cells in different ways and the combination can, on one hand, kill cells with targeted drugs (PARP inhibitors) and cytotoxic drugs (chemotherapy), and on the other hand, augment the neoantigen load and help the immune system to attack the tumor. In this regard, combination therapies are effective in a lot of tumor types [182]. A phase II clinical trial in patients with metastatic PCa is evaluating the therapy with cabazitaxel, carboplatin, and cetrelimab in the first phase, followed by niraparib with or without cetrelimab [183]. The primary endpoint is PFS and the study will be completed in 2025 [183].

### 6.5. Immunocheckpoint Inhibitors in Combination Therapies

Another plausible strategy could be to overcome resistance to mono-immunotherapy by associating an anti-PD1 with an anti-CTLA4.

In CheckMate 650, a Phase II clinical study of the combination of nivolumab and ipilimumab in patients with mCRPC, the ORR was 25% in the experimental treatment [184].

In another phase II study, patients with ARV7 were subjected to a combination of nivolumab and ipilimumab and treated with or without enzalutamide. Overall, 20% (3/15) of patients without enzalutamide and 26.7% (4/15) of patients with enzalutamide achieved a progression-free survival of greater than two years. In the non-enzalutamide arm, the PSA response was 13% and the ORR was 2%, and in the enzalutamide arm, the PSA response rate and ORR were demonstrated to be 0% and 0% [185]. Further ongoing studies are summarized in Table 2.

### 6.6. Immunochechpoint Inhibitors with Vaccine and Other Drugs

Immunotherapy has also been studied in combination with vaccines containing costimulatory molecules.

In a phase I study, Ipilimumab was combined with a vaccine containing transgenes for costimulatory molecules and for PSA in patients with mCRPC (PROSTVAC), and demonstrated a reduction in PSA in 14 of 24 (58%) patients not pretreated with chemotherapy [186].

Pembrolizumab was studied in phase I and II studies in mHSPC newly diagnosed oligometastatic or mCPRC patients in combination with cryotherapy, with ADXS31-142 (a cancer vaccine containing a live attenuated strain of Listeria monocytogenes encoding a PSA fusion protein and a fragment of listeriolysin O) and MVI-816 (a DNA vaccine encoding prostatic acid phosphatase) [161,187,188,189,190].

In a phase I study of 24 mCRPC patients with a fixed dose of GM-CSF with an escalating dose of ipilimumab, the treatment induced the expansion of specific T cells against the tumor and 50% of patients treated with the higher dose had a PSA drop >50% [191].

A combination of ipilimumab with evophosphamide, a pro-drug that relieves hypoxia, resulted in stable disease in 18 patients and in 3 patients (16.7%) with partial responses; all responders had an augmentation of intratumoral T-cell infiltration [192].

Other phase I and II trials in mCRPC patients have investigated the combination therapy of anti-PD-1 with IDO1 inhibitors, anti-IL6, anti-TGF-β, B7-H3 inhibitors enoblituzumab, LAG-3, OX40, and 4-1BBL [193,194,195,196,197] (NCT03821246, NCT-02628535, NCT02923180, NCT01391143).

Further ongoing studies are summarized in Table 2.

## 7. Side Effects of Treatments

All of the therapies mentioned above are not free from side effects. Each treatment has side effects related to the properties of the drug. Immunotherapy is associated with autoimmune side effects such as arthritis, colitis, skin toxicity, hypophysitis, thyroiditis, hepatitis, and renal and cardiological toxicity. However, the use of CAR-T therapies is associated with the risk of even more serious adverse events. In particular, cytokine release syndrome (CRS), which is characterized by the possible occurrence of fever, a drop in blood pressure, an increase in heart rate, chills, and low blood oxygen; these are caused by the intense inflammatory response that develops following the activation of CAR-T cells in the body. In the majority of cases, it occurs within 7–14 days after the administration of the therapy. Patients with a severe CRS require interruption of treatment, and in rare cases, it proves fatal. In cases of severe CRS, specific drugs are administered to reduce the levels of inflammatory substances in the circulation (for example tocilizumab or cortisone). Another adverse event is the reduction in B lymphocytes and antibodies (hypogammaglobulinemia) because CAR-T therapies involve, together with the tumor cells, the destruction of B lymphocytes, with a reduction in the level of antibodies. In some cases, to achieve a normal concentration of antibodies and reduce the infectious risk, it is necessary to subject the patient to periodic infusions of human immunoglobulins. Severe adverse events are related to neurological adverse reactions, and the most commonly observed are CAR-T-cell-related encephalopathy syndrome (CRES), tremor, aphasia, and delirium. Most cases occur within 8 weeks following the administration of the CAR-T therapy. The average duration of these alterations is 14 days, with the complete disappearance of symptoms in 98% of patients [198]. Even classic treatments are not free from side effects [199]: chemotherapy is associated with asthenia, diarrhea, vomiting, pain, fatigue, among the most common side effects, which can often affect quality of life; ARSI is associated with reduced sexual desire, erection difficulties, low number of red blood cells, liver and neurological toxicity; radiotherapy can lead to various side effects, with skin toxicity, lymphedema, and hematological toxicity among the most common. The side effects of the various treatments are added together in combination therapies and, although they are promising from the point of view of survival outcomes, the choice of treatment and lines of research cannot ignore the adverse events in this older patient setting.

## 8. Discussion

Immunotherapy can be an excellent opportunity for patients with prostate cancer for several reasons. For example, it is associated with manageable side effects compared to chemotherapy in this population of often elderly patients, as well as the possibility of having an extra weapon for patients who are not responsive to other treatments. The role of the tumor microenvironment in the poor response of prostate cancer to immunotherapy is well established. The most important questions regarding the resistance mechanisms concern the interaction between the tumor microenvironment and tumor cells, which are governed by various genetic mechanisms. There is a common thread between the composition of the tumor microenvironment and genetic alterations.

Tumor immune evasion is highly correlated with the upregulation of B7 inhibitory molecules in the TME. CD276 is a B7 family member related to impaired TMJ and BRCA function and low tumor infiltrating lymphocytes (TILs), and it is closely related to PTEN deficiency [87,88,89]. In this interpretation, it is clear how modifying the genetics of PCa would also lead to a modification of the tumor microenvironment. Combination therapies are hopeful as, by combining the different treatments, it would seem possible to overcome the resistance. An important aspect is also related to costs; for example, immunotherapy with sipuleucel-T added to standard treatment led to an increase in quality-adjusted life years (QALYs) of 0.37, with an additional cost of around USD 104,536. In this regard, the incremental cost–utility ratio, which is important for estimating costs, was USD 283,000 per QALY saved. These data demonstrate the high costs of immunotherapy in prostate cancer [200]. We believe in immunotherapy as a great opportunity [201]; however, the costs of this treatment should be balanced with its effectiveness. In this regard, the most promising lines of research on immunotherapy must, above all, take into account patient selection: the real key to balancing the costs with the benefits and side effects would be to identify a defined subgroup of patients who respond well to each treatment. This is, from our perspective, the research key to be associated with trials on combination therapies.

## 9. Conclusions

Immunotherapies have transformed oncology and improved the outcome of a lot of cancer types. This approach allowed us to achieve durable immune control, but it seemed to be less effective in PCa patients. PCa has been considered an immunologically “cold” tumor because of the immunosuppressive characteristics of the TME. The high hopes about immunotherapy in PCa did not initially receive confirmation. This failure has stimulated scientific research to investigate the composition of the TME of PCa and possible solutions to modify it and make it sensitive to immunotherapy in the future. Various combination therapies are underway and this strategy is promising in this setting of patients. These approaches aim to transform the PCa TME from “cold” to “hot” in order to improve strategy and rewrite the treatment of PCa.

## Figures and Tables

**Table 1:** Studies evaluating single agent immune checkpoint inhibitors (ICI) in CRPC.

Trial Name	Phase	Treatment Plan	Setting	Primary Endpoints	Secondary Endpoints	Results
Ipilimumab						
Pilot study of Ipilumumab [122]	I	3 mg/kg i.v. dose of Ipilimumab	mCRPC	Serologic measures of autoimmunity, evaluation of T-cell activation	Pharmacokinetic sampling of plasma for MDX-CTLA-4, PSA measurement	-No objective response -Biochemical response. -Immune adverse events limited to one patient.
CA184-095 [123]	III	High-dose (10 mg/kg) ipilimumab monotherapy or ipilimumab dose escalation vs. placebo	mCRPC	OS	PFS, safety	-High dose did not reached OS over placebo (28.7 months vs. 29.7 months; HR = 1.11, 95% CI 26.1–34.2 months, *p* = 0.3667). -Dose escalation lengthened median PFS (5.6 months vs. 3.8 months; HR = 0.67, 95.87% CI 0.55–0.81), decreased of PSA levels (23% vs. 8%). Incidence of grade 3–4 adverse reactions and treatment-related deaths increased.
CA184-043 [124]	III	CRPC bone metastases progressing after docetaxel therapy were randomized to receive bone-targeted radiotherapy followed by ipilimumab 10 mg/kg or placebo	CRPC with bone metastasis	OS	PFS, safety	Not reached
Pembrolizumab						
KEYNOTE-028 [125]	Ib	Pembrolizumab in pretreated mCRPC patients with measurable disease and PD-L1 expression ≥1% in tumor or stromal cell	Incurable advanced biomarker-positive solid tumors	ORR	OS, PFS, DOR	-ORR of 17.4 (4 patients with partial response, 3 of them with biochemical response) -Safety
KEYNOTE-199 [126]	II	Pembrolizumab monotherapy in 3 patient cohorts: PD-L1 positive tumor and measurable disease, PD-L1 negative tumors and measurable disease, and non-measurable metastatic bone disease regardless of PD-L1 status.	mCRPC	ORR	Safety, DCR, DOR, PSA response rate, PFS, OS, Duration of PSA Response and others	-OS and ORR were modest. -Activity in both RECIST measurable diseases and bone-predominant diseases, regardless of PD-L1 expression.
Atezolizumab						
PCD4989g [130]	I	Atezolizumab was given intravenously every 3 weeks until confirmed disease progression or loss of clinical benefit.	MCRPC who had progressed on sipuleucel-T or enzalutamide	Safety	Efficacy, biomarker analyses,	-Good safety profile,—Overall survival rate of 12 months survival of 55.6% and -Six-month progression-free survival rate of 26.7%.
Avelumab						
JAVELIN solid tumor [131]	I	Avelumab, multiple-ascending dose Trial	Metastatic or locally advanced Solid Tumors and expansion to selected indications	Safety, Tolerability, Pharmacokinetics, Biological and Clinical Activity	Efficacy Expansion Cohort	-10 mg/kg dose was chosen as the most effective in an expansion cohort of 18 mCRPC. -7 patients: stable disease after 24 weeks of treatment -6 patients: disease progression at six weeks.

**Table 2:** Combination therapy trial ongoing.

Trial Name	Phase	Treatment Plan	Setting	Primary Endpoints	Secondary Endpoints
Combinations of ICI’s					
INSPIRE (NCT04717154)	II	Ipilimumab, Nivolumab	mCRPC	Disease control rate (DCR)	Safety, ORR, BRR, PFS per irRECIST1.1 immune-related response criteria
IMPACT (NCT03570619)	II	Ipilimumab, Nivolumab	mCRPC and CDK12 mutations	ORR, Response will be defined as a 50% decline in PSA (prostate specific antigen) from baseline as determined by PCWG3 criteria.	RPFS, PFS, DOT, TTP, OS, PSA-PFS, Time to PSA progression
NCT05293496	I	MGC018 (CD276 inhibitor), lorigerlimab (dual PD-1 × CTLA-4 inhibitors)	mCRPC and other tumors	Aes, SAEs	Cmax, Tmax, AUCtau, Trough concentration of vobramitamab duocarmazine and lorigerlimab, ORR, PFS, DoR, OS, rPFS, PSA-response rate, Best PSA percent change, and others
NCT03061539	II	Nivolumab, Ipilimumab	PCa	Composite response rate: Radiological response (RECIST 1.1), PSA response ≥50% confirmed by a second PSA test at least 4 weeks later, Conversion of CTC count from ≥5 cells/7.5 mL at baseline to <5 cells/7.5 mL confirmed by a second CTC test at least 4 weeks later	OS, PFS, PSA-PFS, Change in patient reported outcome measures, safety
NCT03651271	II	Nivolumab, Ipilimumab	PCa and other tumors	CBR, Percentage of CD8 cells in on-treatment biopsies	Safety, ORR, The association of the percentage of CD8 cells in tumor samples with clinical outcomes
ICI’s and chemotherapy					
NCT04100018	III	Nivolumab, Prednisone, Docetaxel	PCa	rPFS, OS	ORR, DOR, PSA Response Rate (PSA-RR), Time to PSA Progression (TTP-PSA), safety, median time to pain progression
NCT05169684	II	BMS986218 (CTLA4 inhibitor), Docetaxel, Nivolumab	mCRPC	Safety, number of deaths, rPFS	Objective response rate, time to response, duration of response, PSA-RR, TTP-PSA, OS, safety, number of deaths
ICI and adoptive cell therapy/vaccines					
NCT03406858	II	Pembrolizumab, HER2Bi-armed T cells	PCa	PFS	
NCT03792841	I	Acapatamab (bispecific T-cell engager), Pembrolizumab	PCa	Safety, Number of participants with dose-limiting toxicity	Cmax and minimum serum concentration of acapatamab, AUC over the dosing interval of acapatamab, OR, PSA-RR, DOR, Percentage of participants experiencing a response based on 68Gallium (68 Ga)-prostate-specific membrane antigen (PSMA)-11 positron emission tomography (PET)/computed tomography (CT) response evaluations, Percentage of participants experiencing a response based on 18F-fluorodeoxyglucose (FDG) positron emission tomography (PET)/computed tomography (CT) response evaluations, PFS, OS, and others
NCT02933255	I/II	PROSTVAC-V/F, Nivolumab	PCa	Safety, Evaluate changes in T-cell infiltration in the tumor after neoadjuvant treatment	Safety, Changes in soluble immune mediating factors, Changes in PDL-1 expression, Changes in immune cell subsets, changes in circulating tumor cells, Pathologic responses, PSA changes, and others
Other combinations					
Rad2Nivo (NCT04109729)	Ib/II	Nivolumab, Radium-223	mCRPC	Safety of this combination treatment, then expand into a phase II cohort to assess the ctDNA reduction after 6 weeks of nivolumab treatment.	PSA-PFS, correlation of bone metabolism markers with clinical response, response rates by serum PSA, time to first symptomatic skeletal related event, and others
NCT04159896	II	ESK981 (multi-tyrosine kinase inhibitors), Nivolumab	mCRPC	PSA ≥ 50% response rate (PSA50), safety	Time to PSA response (TTPR)
PORTER (NCT03835533)	I	NKTR-214 (CD122-preferential IL2 pathway agonist), Nivolumab, SBRT, CDX-301 (FLT3 ligand, a dendritic cell mobilizer), INO-5151 (combination of DNA plasmids encoding IL-12 and PSA/PSMA)	PCa	Safety	ORR, Disease control rate, rPFS, OS
STELLAR#001 (NCT03845166)	I	XL092 (tyrosine kinase inhibitor that targets VEGF receptors, c-Met), Atezolizumab, Avelumab	mCRPC and other tumors	Recommended dose for XL092, ORR, PFS, OS	Aes, SAEs, Tmax, Cmax, AUC 0–24, Terminal Half-Life, Apparent Clearance (CL/F)
IceCAP (NCT03673787)	I/II	Ipatasertib (AKT inhibitor), Atezolizumab		Proof of concept for the combination of ipatasertib and atezolizumab acting on PI3K hyperactivated tumors	
IceCAP (NCT03673787)	I/II	Ipatasertib (AKT inhibitor), Atezolizumab	mPCa and other tumors	Maximum tolerated dose in Phase I, number and type of Aes of the two-drug combination	Changes in immune-cell population in blood and plasma, assess the tumor microenvironment by immunophenotyping
